# Accounting for equity considerations in cost-effectiveness analysis: a systematic review of rotavirus vaccine in low- and middle-income countries

**DOI:** 10.1186/s12962-018-0102-2

**Published:** 2018-05-18

**Authors:** Marie-Anne Boujaoude, Andrew J. Mirelman, Kim Dalziel, Natalie Carvalho

**Affiliations:** 1 Faculty of Economics, University of Bologna, Bologna, Italy; 2 Centre for Health Economics, University of York, York, UK; 3 Centre for Health Policy, The University of Melbourne, Melbourne, Australia; 4 Centre for Health Policy & Global Burden of Disease Group, School of Population and Global Health, University of Melbourne, Melbourne, Australia

**Keywords:** Cost-effectiveness analysis, Equity, Health equity, Rotavirus vaccine, Low- and middle-income countries

## Abstract

**Background:**

Cost-effectiveness analysis (CEA) is frequently used as an input for guiding priority setting in health. However, CEA seldom incorporates information about trade-offs between total health gains and equity impacts of interventions. This study investigates to what extent equity considerations have been taken into account in CEA in low- and middle-income countries (LMICs), using rotavirus vaccination as a case study.

**Methods:**

Specific equity-related indicators for vaccination were first mapped to the Guidance on Priority Setting in Health Care (GPS-Health) checklist criteria. Economic evaluations of rotavirus vaccine in LMICs identified via a systematic review of the literature were assessed to explore the extent to which equity was considered in the research objectives and analysis, and whether it was reflected in the evaluation results.

**Results:**

The mapping process resulted in 18 unique indicators. Under the ‘disease and intervention’ criteria, severity of illness was incorporated in 75% of the articles, age distribution of the disease in 70%, and presence of comorbidities in 5%. For the ‘social groups’ criteria, relative coverage reflecting wealth-based coverage inequality was taken into account in 30% of the articles, geographic location in 27%, household income level in 8%, and sex at birth in 5%. For the criteria of ‘protection against the financial and social effects of ill health’, age weighting was incorporated in 43% of the articles, societal perspective in 58%, caregiver’s loss of productivity in 45%, and financial risk protection in 5%. Overall, some articles incorporated the indicators in their model inputs (20%) while the majority (80%) presented results (costs, health outcomes, or incremental cost-effectiveness ratios) differentiated according to the indicators. Critically, less than a fifth (17%) of articles incorporating indicators did so due to an explicit study objective related to capturing equity considerations. Most indicators were increasingly incorporated over time, with a notable exception of age-weighting of DALYs.

**Conclusion:**

Integrating equity criteria in CEA can help policy-makers better understand the distributional impact of health interventions. This study illustrates how equity considerations are currently being incorporated within CEA of rotavirus vaccination and highlights the components of equity that have been used in studies in LMICs. Areas for further improvement are identified.

**Electronic supplementary material:**

The online version of this article (10.1186/s12962-018-0102-2) contains supplementary material, which is available to authorized users.

## Background

Equity constitutes an integral aim of public health policies worldwide. The importance of addressing health inequities as a goal in the health sector in both developing and developed countries was explicitly stated in the Declaration of Alma-Ata in 1978 [1]. This focus has translated into the academic arena, with 216% more articles published in MEDLINE having the word equity in their abstracts in 2015 compared to those published in 1980 [2]. From a policy perspective, equity is at the heart of the United Nations’ 2030 Agenda for Sustainable Development, with several task forces and committees established to work towards equity goals.

Whether referred to as fairness or social justice, health equity alludes to the fair distribution health. It concerns the differences in population health that can be traced to unequal economic or social conditions [3]. According to Culyer’s interpretation of Aristotle, equity can be distinguished as horizontal and vertical equity: horizontal equity entitles like treatment for like individuals and vertical equity unlike treatment for unlike individuals in proportion to the differences between them [4]. A range of methods have been proposed to quantify the magnitude of health inequity, including rate ratios, population attributable risks, slope and relative indices of inequality, and the concentration curve and index [5].

Given the limited resources available to fund health systems, cost effectiveness analysis (CEA) is a useful tool to guide the allocation of health budgets. Decision makers are simultaneously seeking to achieve diverse goals such as: maximizing health, reducing health inequities and providing protection against the costs of ill health [6]. CEA results provide evidence on how to maximize health benefits within a given budget, accounting for the societal value of health. CEA, however, does not generally provide information about the distributional value of health benefits in a given setting [7]. In fact the CHEERS guideline does not mention equity as an item to include when reporting economic evaluations of health interventions [8]. Despite this limitation, many guidelines suggest that social value judgments can be implicitly incorporated in CEA by choosing which parameters to include in the analysis [9] or by adhering to certain principles such as those in the Gates Reference Case [10]. The Guidance on Priority Setting in Health Care (GPS-Health) developed by Norheim et al. in 2014 constitutes an explicit guidance on the inclusion of fairness in the decision-making process. It lays out a set of criteria to go beyond health maximization as reflected by traditional cost-effectiveness alone by providing a list of the equity-relevant dimensions that can be explicitly integrated in CEA.

Previous systematic reviews, studies and guides have proposed many approaches to integrate equity concerns into CEA. Earlier reviews, mainly Sassi et al. [11] and Hauck et al. [12], report approaches to weight health outcomes in different subgroups along specific equity dimensions. Specific weights are developed using a willingness-to-pay measure or person trade-off technique to weight cost-effectiveness ratios for different indicators (e.g. severity) and age weighting functions have been used to weight DALYs. Inequalities in non-health factors (economic situations, political positions or occupational groups) are mentioned as important to be taken into account when deriving equity weights. More recent reviews and studies, Johri et al. [7] and Cookson et al. [13], discuss the latest thinking on methods to address equity concerns in CEA, such as accounting for the distribution of opportunity costs, the use of mathematical programming, multi-criteria decision analysis, and two recently developed approaches: distributional cost-effectiveness analysis (DCEA) and extended cost-effectiveness analysis (ECEA). DCEA, developed by researchers at the University of York, involves two steps: first the modelling of the overall social distribution of health in a setting and that associated with each intervention; and second, it evaluates the gains in health equity (or distributional fairness) as it trades off with total gains in health [13, 14]. The second methodological framework, ECEA, was developed through the Disease Control Priority Network (DCPN) project [15]. This approach accounts for the distributional health consequences in addition to assessing the financial risk protection (FRP) benefits of households, or the prevention from illness-related impoverishment [16]. FRP, one of the outcomes measured in ECEA, quantifies the number of poverty cases averted. Illness-related loss of income and expenditure to seek care are the main causes of financial risks that can be prevented either by preventing the illness or its progression or by having a well-structured health care system [17]. DCEA has mostly been applied to the high-income country setting of the United Kingdom, while ECEA has generally been used for low- and middle-income countries where there is a higher risk of suffering from disease-related impoverishment, although each approach could be applied to either setting. Most reviews conclude that the established methods are either not commonly used or not fully satisfactory. Further noted, challenges that were highlighted were the selection of “equity-relevant” characteristics for each setting and disease and the difficulty of prioritizing those characteristics.

The purpose of this review is two-fold. Firstly to develop a comprehensive list of equity-relevant indicators based on the equity criteria contained within the Guidance on Priority Setting in Health Care (GPS-Health) by Norheim et al. [18]. The GPS-Health checklist consists of broad criteria that are theoretical rather than operational. Thus, our aim was to use this checklist to develop specific equity indicators that could be operationalized to help researchers and decision-makers navigate through equity-relevant characteristics that can be incorporated in CEAs. Secondly to assess the extent to which the mapped indicators were incorporated in the analysis of published CEAs, and whether they were reflected in the results or also included as an explicit equity objective of the study. It provides a form of assessment tool to evaluate equity in existing cost-effectiveness studies.

We chose to focus our study on cost-effectiveness analyses of rotavirus vaccines in low- and middle-income countries. The use of CEA has been central in many countries to decisions regarding the introduction of vaccines in national immunization programs. The adoption of second wave vaccines such as rotavirus, human papillomavirus (HPV), pneumococcal conjugate, Hepatitis B, and *Haemophilus influenzae* type B vaccines have been less straightforward than traditional childhood vaccines. Compared to the first wave of mass vaccination (vaccines against measles, diphtheria, tetanus and pertussis), the second-wave vaccines are more costly and are less consistently cost effective [19]. Consequently, the costs and benefits of new vaccines must be carefully weighed, especially in low-resource settings [14]. Understanding the equity impacts of new vaccine introduction is also an important consideration. For example, Gavi, the Global Alliance for Vaccines and Immunizations 2016–2020 strategy addresses within-country inequities in immunization through the use of an equity indicator measured as the difference in coverage between the wealthiest and poorest wealth quintiles of a country [20].

Rotavirus vaccination, recommended by the World Health Organization for all countries, has witnessed a partial uptake in LMICs despite demonstrated cost-effectiveness [21]. Two vaccines are currently licensed for the prevention of rotavirus, which accounts for 35–55% of gastroenteritis, 40% of diarrhea-related hospitalization in children aged less than 5 years and has a mortality rate of 3.4% worldwide [22, 23]. The choice of introducing rotavirus vaccine, or any vaccine, in a LMIC is usually driven by complicated decision process including considerations such as disease burden, concerns about healthcare spending, and vaccine program costs. So far, rotavirus vaccine has been introduced in 93 countries and 23 are planning its introduction. The 76 remaining countries have not introduced the vaccine yet and account for 32% of the population worldwide [24].

Results of this study offer important insights into the equity indicators relevant for vaccine CEAs, in addition to reviewing the extent to which indicators are incorporated within CEA.

## Methods

The methodology of this study has three parts: mapping of equity indicators, systematic literature search and the assessment of the selected studies with regards to their incorporation of equity.

### Equity indicators mapping

The GPS-Health checklist [18] (Additional file 1: Appendix A) was developed via a thorough search of the literature and a series of consultations and is a recent and comprehensive guidance for looking at equity in health priority setting. Joining the point of views of both scientists and decision makers, the criteria in the checklist were divided into three categories: *disease and intervention criteria*, *criteria related to characteristics of social groups, criteria related to protection against the financial and social effects of ill health*.

**Fig. 1:**
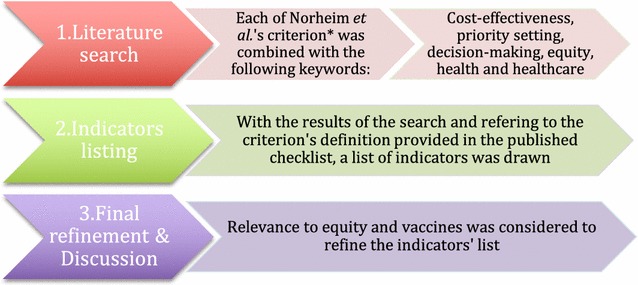
Steps of indicators’ mapping. *Norheim et al.’s criteria being: Severity, realization of potential, past health loss, socioeconomic status, area of living, gender, race, ethnicity, religion, sexual orientation, economic productivity, care for others and catastrophic health expenditure

Each of the ten criteria was entered as search keywords and combined with the following additional keywords: cost-effectiveness, priority setting, decision-making, equity, health and health care. Pubmed/MEDLINE and EconLit were used and the exercise was carried out in February 2017. The process is summarized in Fig. 1 and the search strategy is in Additional file 2: Appendix B. The articles identified were screened for the inclusion of the indicator or a related indicator and methods were interpreted. As a first step, for each criterion, candidate indicators of equity were selected and listed in light of the definitions and examples provided by the original checklist. After developing this list, the catalogued indicators were reviewed and discussed by NC, MB and KD for specific application to vaccines, rather than more broadly for all interventions, as outlined in the original checklist. In a third step the indicators were subjected to a final selection through discussion amongst the authors: NC and MB, and then by NC, AM and KD. The indicators deemed not related to equity as applied to vaccines were removed. A refinement followed to adapt the indicators to childhood vaccines for this particular case study. Productivity loss, for example, was designated as productivity loss of caregivers instead of patients to make it applicable to childhood vaccines.

### Systematic review

We used studies identified from a published systematic review on economic evaluations of rotavirus vaccine in low- and middle-income countries by Carvalho et al. [25]. From the studies included in the final review (n = 66), the economic evaluations described as CEA were retained (n = 60) and the rest were discarded (n = 6).

Although equity has not been considered an integral component of CEA until very recently with the new methodological developments, important reviews on the concepts and principles of equity in health [11, 26] have been published in the 20th century. By selecting a time frame between 2000 and 2017, we aimed to cover the entire period in which equity could have been taken into account in CEAs. Studies were included if they met the following inclusion criteria: (1) published between January 2000 and February 2017, (2) peer reviewed articles (3) focused on one or more LMICs, (4) target population of children under 5, (5) intervention is any rotavirus vaccine delivered in any manner.

Duplicate citations were removed and all remaining papers were screened based on title and abstract. Non-English language papers included after abstract review were translated to English (n = 4). We followed the PRISMA (Preferred Reporting Items for Systematic Reviews and Meta-analysis) guidelines and checklist for the review [27].

### Data extraction

Studies included in the systematic review were assessed for the incorporation of equity considerations based on the indicators identified in the first step of the methods. In a first screening, for each equity indicator, we considered the following: (1) whether the article mentions the indicator; (2) whether the article incorporates the indicator in its analysis and results, and (3) whether the indicator is neither mentioned nor incorporated. A second screening focused on how the indicators were included in the study: (1) indicators included in input, (2) indicators shown in output, (3) equity framed in study objective. The input refers to the parameters included in the evaluation while the output deals with the results of the evaluation. Framing equity refers to having in the study an explicit statement of integrating distributional concerns in the analysis. A descriptive analysis followed.

Screening was performed by one reviewer (MB). A consistency check was performed by second reviewer (NC) who extracted data independently from a 10% random sample of articles from the 60 studies identified.

## Results

### Attribution of indicators

The equity indicators developed are summarized alongside the general GPS-Health checklist from Norheim et al. [18] in Table 1.

**Table 1:** Summary of the GPS-Health checklist and mapped indicators

GPS-Health Equity checklist^a^	Indicators^b^	Definition
Group 1: Disease and intervention criteria		
Severity	Severity of illness at the individual level	Mild/moderate/severe
	Age distribution of the disease	Disease incidence by age group
Realization of potential	Final health status	Health status after treatment showing treatment benefit capacity
Past health loss	Presence of comorbidities	Presence of other diseases or conditions with the studied disease
Group 2: Criteria related to characteristics of social groups		
Socioeconomic status	Household income level	Wealth level, occupation, and socioeconomic indices
	Relative coverage	Difference in coverage between the richest and poorest quintile
	Education	Education level of patients or parents
Area of living	Geographic location	Urban/rural; by province or by state
Gender	Sex at birth	Male/Female
Race, ethnicity, religion and sexual orientation	Race	Having certain physical characteristics
	Ethnicity	Belonging to a social group
	Religion	Belonging to a religious group
	Sexual orientation	Homo/Heterosexual
Group 3: Criteria related to protection against the financial effect of ill health		
Economic productivity	Loss of productivity	Productivity lost due to illness
	Age	Age-weighting of DALYs
Care for others	Number of dependent persons	Are children or elderly depending on the patient?
Catastrophic health expenditure	Financial risk protection	Protection against catastrophic spending
	Reliance on OOP expenditure	OOP spending due to illness for treatment or care

^a^GPS-Health Equity checklist developed by Norheim et al. [18]

^b^Indicators developed by authors

#### Disease and intervention criteria

This group was formed of three criteria: (1) Severity, (2) realization of potential, and (3) past health loss.

Severity was assigned two indicators: firstly, severity of illness at the individual level, commonly considered as a measure to reflect the level of need, conveys that for some treatments different disease severities would incur different costs and results in different benefits [28]. Secondly, age distribution of the disease, which reflects differences in severity according to age: children less than 6 months of age are protected by maternal antibodies and those between 6 and 12 months are more likely to be hospitalized due to rotavirus infections [29]. Including an age distribution of rotavirus illness in the study for each of the disease outcomes (e.g. out-patients, hospitalizations and deaths) accounts for the different possible scenarios according to age. Different age groups have different risks of illness and deaths, and incur diverse costs. Final health status was the indicator attributed to realization of potential. It reflects the capacity or potential to benefit from a certain treatment or intervention [30, 31].

The third criteria, past health loss, was attributed the indicator presence of comorbidities, whether chronic diseases or other health conditions. It highlights the potential special value of an intervention if it targets a group that has suffered significant past health loss [32].

#### Criteria related to characteristics of social groups

Four criteria were presented in this group:(1) Socioeconomic status, (2) Geographic location, (3) Gender, (4) Race, Ethnicity, Religion and Sexual orientation.

Three indicators were mapped to Socioeconomic status: Household income, Education and Relative coverage. Household income—encompassing wealth level, occupation, tenure and socioeconomic indices—and Education are two relevant indicators of socioeconomic position related to health and vaccination status. Education, at the individual level, is a relevant factor for vaccination as it impacts parents’ decision and efforts to vaccinate their children [33]. Relative coverage was highlighted as Gavi’s principal equity indicator and a measure used to reflect the extent to which a certain programs’ coverage reaches those at higher risk. Relative coverage is a “derived indicator” based on wealth ratios and is calculated by the differences in coverage between the richest and the poorest quintiles [34, 35].

Area of living refers to the geographic location and captures the contribution of geographic inequalities in health and access to health care [36]. This indicator considered rural/urban differences or state/provinces boundaries, depending on the study. Race, Ethnicity, Religion and Sexual orientation were separated into four disparate indicators: Race, Ethnicity and Religion to acknowledge that some disadvantaged groups might need to be given a certain level of attention and priority [37] and Sexual orientation was found to be particularly relevant for a certain set of diseases (e.g. sexually transmitted diseases) [38].

#### Criteria related to protection against the financial and social effects of ill health

This group’s three criteria (8) Economic productivity, (9) Care for others, and (10) Catastrophic health expenditure were assigned five indicators. Two indicators were distinguished for Economic productivity: The first indicator is Loss of productivity, whether it relates to the informal caregiver’s productivity or patient’s productivity, it has an important financial impact on the household [39]. Loss of productivity is measured through the foregone or incremental income, absenteeism and presenteeism. The second is Age, accounting for average age of the population benefiting from the treatment (e.g. treatment targeting children, adults or retired). DALYs age-weighting represent, to a certain extent, the economic productivity of an individual, given very young and older ages depend physically, emotionally and financially on individuals belonging to the “economically productive” ages. They are thus assigned lower weights [40, 41]. A continuous debate revolves around this indicator: critics question why age only was given an important social value and not any other socioeconomic component. Moreover, Years of life lost (YLL) favors the young, so age-weighting DALYs would only double this emphasis [42]. It is worth mentioning that the WHO cost-effectiveness guidelines published in 2000 presented its result with and without age weighting and the one published in 2010 omitted the inclusion of age weighting [43]. The criterion “Care for others” was assigned the indicator “Number of dependent persons” (e.g. children or elderly). The value of the intervention might increase with the increase of the number of persons depending on the patient. Two indicators were mapped to Catastrophic health expenditure: The Reliance on of Out-Of-Pocket (OOP) expenditure and Financial Risk Protection (FRP), both of major relevance in LMIC’s which may have poor coverage of health insurance [44]. The reliance of OOP expenditure reflects the weight of reliance on private funds to pay for an intervention. It can be OOP as a percentage of consumption, or the quantification of the amount spent OOP on certain health services.

Financial risk protection addresses illness-related impoverishment. It measures the extent to which an intervention protects a household from catastrophic expenditure leading to poverty and reflects inequalities in income and wealth [45].

### Articles included in the systematic review

**Fig. 2:**
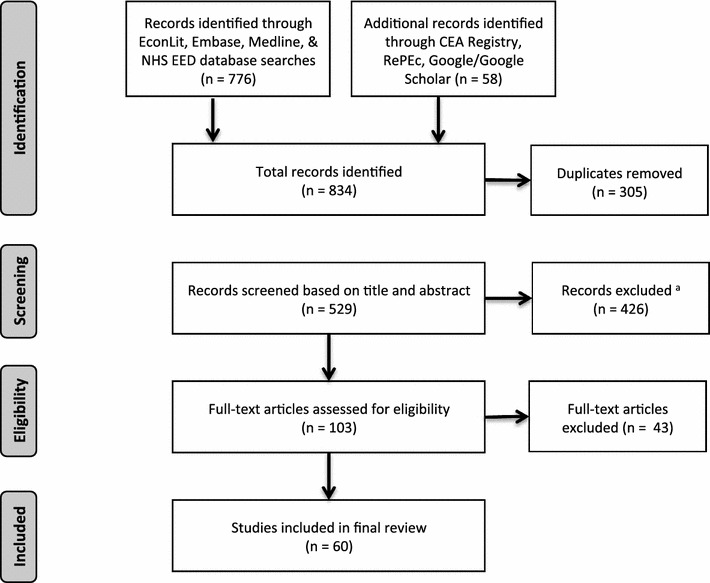
Literature search flow diagram. Flow diagram adapted from “Capturing Budget Impact Considerations Within Economic Evaluations” by Carvalho et al. [25]. a Including one article in Russian for which full text could not be obtained

The study selection process is presented in Fig. 1. A total of 529 articles were identified after duplicate removal and screened for inclusion and exclusion criteria based on title and abstract. 103 full text studies were read by two reviewers [25] and 44 articles were excluded. In the final review, 60 CEAs were included (Fig. 2).

The articles were published between 2005 and 2016, with rotavirus vaccine introduction starting in 2006. 52 articles (87%) focused on individual LMICs around the world and 8 (13%) studied groups of countries. Even though a significant portion of studies focused on Asian countries (23 articles—44% of the single country articles), the continent has the lowest uptake of rotavirus vaccine [46].

### Application of indicators to rotavirus vaccine

The articles were assessed by MB and the consistency check run by NC resulted in 97% agreement across all categories.

Of the 18 mapped indicators, two were judged to be less relevant to the specific case of rotavirus vaccine due to the disease characteristic and the intervention’s targeted population: (1) Final health status: the sick children either died or recovered from the infection, no other health status could be attained and (2) Number of dependent persons: being a childhood vaccine, the individuals contracting the virus are not caring for any other persons but are cared for; and five indicators were not taken into account in any of the articles: (3) Education, (4) Race, (5) Ethnicity, (6) Religion, (7) Sexual Orientation. The eleven remaining criteria were relevant and appeared in the identified articles.

#### Severity

Severity of illness at the individual level was taken into account in 45 (75%) of the included articles by categorizing cases into three severity levels: mild, moderate, and severe. The levels were defined in terms of degree of care needed, whether no care, outpatient care or hospitalization respectively was required. The results were all presented as events and costs averted per degree of severity. The remaining 15, forming 25% of the studies, did not capture differing levels of disease severity. It is worth mentioning that 3 (20%) of these remaining studies were Extended Cost-Effectiveness Analysis, 5 (33%) constituted studies dealing with groups of countries (whether Gavi-eligible countries or developing countries) and only 7 (47%) were single-country studies.

#### Age distribution of the disease

Age distribution was incorporated in 42 articles (70%). In addition to incorporating it in the input, two studies (3%) reflected the differences in the output as cases averted per age. An example can be found in Martí et al. [47].

#### Presence of comorbidities

Two comorbidities were mentioned within the rotavirus CEAs: malnutrition, mentioned nine times (15%), and the presence of other diseases, mainly pneumonia and HIV, referred to twice (3%).

Malnutrition was incorporated in the analysis of 3 articles (5%). Two articles used proxies to estimate the distribution of rotavirus mortality across wealth quintiles representing higher physical susceptibility as measured by weight for age Z scores [48, 49]. The third developed an evidence-based individual risk index to estimate the relative distribution of mortality within the region-sex populations based on nutritional status and access to basic care for diarrheal disease.

The presence of chronic diseases also affects the input parameters. Two articles (3%) acknowledged special conditions of sub-groups and thus excluded children with pneumonia or HIV from their analysis.

#### Household income level

Household income was mentioned since 2006 as an important criterion to be taken into account when performing CEA, but it is not until 2012 that studies began dividing their results per wealth quintile. 5 (8%) of the identified articles presented their results accordingly. Results, in terms of incremental cost-effectiveness ratios (ICERs) and deaths averted, from the same country were quite different between wealth quintiles: the richest quintiles had significantly higher ICERs per DALY averted and the poorest had more deaths averted with the implementation of the intervention. The Rheingans study [49] illustrates these differences: the richest quintile’s ICER (180$ per DALY averted) in India was more than triple the poorest quintile’s ICER (55$ per DALY averted).

#### Relative coverage

**Fig. 3:**
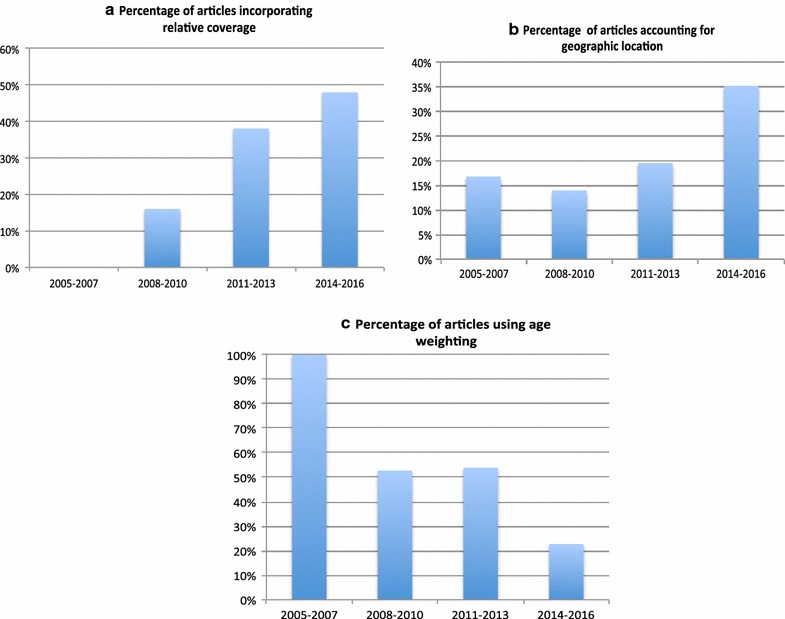
Trends over time in the number of articles incorporating **a** relative coverage, **b** Geographic location, and **c** age weighting. *Percentage of articles out of total number of articles published during the specified period of time

An adjustment factor for relative coverage was applied to the coverage estimates to account for the likelihood that children at the highest risk of dying from rotavirus disease are less likely to be vaccinated [50]. The inclusion of relative coverage in the CEAs increased over time (Fig. 3a) with a total of 18 articles from the 60 (30%) including this indicator. Clarke et al. [51] demonstrated that relative coverage is a key driver in CEA through sensitivity analyses (along with the herd effect multiplier).

#### Geographic location

16 (27%) of the identified articles included geographic location in the analysis. For some countries, important differences in ICERs were detected between different provinces. The inclusion of this indicator was noted to increase over time (Fig. 3b).

Two approaches were used to incorporate the geographic measure: Out of the 27% taking into consideration geographic location, 13 (81%) of the articles accounted for differences in costs between rural and urban areas and some generated weights for that purpose. The remaining 3 articles (19%) took a step further to incorporate (along with costs) incidence and coverage variations. This is clearly shown in Rheingans et al. [52] where the costs, benefits and ICERs were calculated for every region of India.

#### Sex at birth

Sex at birth (male/female) was only incorporated a few times (5%). Wilopo et al. [53] assumed different rotavirus-specific mortality rates for males and females. Megiddo et al. [33] incorporated a stochastic function based on several characteristics (one of which is sex) by which children contract the disease and Rheingans et al. [54] modeled a unit of analysis defined as equal to: geographic area × wealth quintile × sex.

#### Loss of productivity

In the childhood vaccines setting, the productivity loss accounted for is that of the caregiver. Caregiver productivity was often mentioned in the methods (44 times; 73%), however only 27 articles (45%) incorporated the caregiver’s lost income in the societal costs.

#### Age

The impact of age on productivity was taken into account by age weighting of DALYs. 26 articles (43%) age weighted DALYs but this method is shown to be decreasing over time (Fig. 3c).

#### Financial risk protection (FRP)

Health gains and FRP were taken into account in all of the evaluations categorized as extended cost-effectiveness analysis (ECEA), given it is an integral part of the approach. Within the rotavirus CEA articles, the importance of financial risk protection was acknowledged in 2006 by Isakbaeva et al. [55] but the actual incorporation of FRP is relatively new and was performed in one study in 2013 [56] and two others in 2015 [48, 57].

#### Reliance on OOP expenditure

Out of 60 articles, 35 (58%) considered a societal perspective and included in the costs’ calculations the OOP expenditure incurred by the patients’ households. The costs averted were separated between government and societal perspective.

#### Equity framing in study objective

**Fig. 4:**
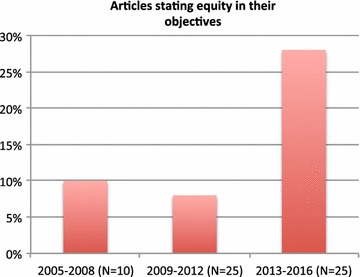
Equity concern framed in study objective over time. *Percentage of articles out of total number of articles published during the specified period of time

The assessment conducted showed that, out of the selected articles, all incorporated at least one equity indicator as a parameter of the cost effectiveness analysis and 54 (90%) articles incorporated at least two. However, not all the studies explicitly mentioned equity as a study objective. Out of all the selected articles, only 10 (17%) explicitly mentioned equity in their study objective, with an increasing trend over time: 10% out of studies published between 2005 and 2008 and 28% out of those published between 2013 and 2016 explicitly had an equity objective (Fig. 4).

Overall, 6 articles stated that they considered the differences between urban and rural communities with the aim of achieving higher levels of equity, 3 articles considered wealth quintile, 3 financial risk protection, 1 societal perspective and 1 sex at birth. Also, 3 articles mentioned distributional consequences as a general objective not related to a specific indicator.

#### Incorporation of indicators in input and output

Indicators were all incorporated in economic evaluation inputs, but in 48 articles (80%), they were also reflected in the economic evaluation results. Results were differentiated with regards to 6 indicators: severity of illness, reliance on OOP expenditure, FRP, household income, age distribution of the disease and geographic location. Severity of illness, shown in the output of 33 articles (55%), divided the costs per level of severity: per outpatient visit, per hospitalization and per death. Reliance on OOP expenditure, shown in the output of 23 articles (38%), provides ICERs for a societal perspective and/or the OOP expenditure averted per infection episode. For household income, 4 articles (7%) present results differentiated by wealth quintile, such as rotavirus cases or deaths averted, private expenditure averted or ICER ($/DALY) averted by wealth quintile. FRP constitutes a specific case as it is designed to be part of the outcome. It focuses on the number of cases of poverty averted instead of costs per health gains or per death averted. Geographic location (in 3 articles—5%) categorizes the results by state, province or urban/rural. Lastly, Age distribution of the disease, shown in the outcome of 2 articles (3%), shows the number of rotavirus infection cases averted by age.

Results are summarized in Table 2, the full data table can be found in Additional file 3: Appendix C, and the proportion of each indicator included in the articles over time in Additional file 4: Appendix D.

**Table 2:** Summary of findings

Indicator	Number incorporating the indicator into analysis^a^	Indicator framed in study objective^a^	Summary of methods used and example	Number of articles incorporating equity indicator in outcome^a,b^
Severity of illness at the individual level	45 (75%)	0	Results split into severity subgroups Example: number of events and costs averted for inpatients and outpatients	33 (55%)
Age distribution of the disease	42 (70%)	0	Including an age distribution function showing the incidence and costs by age	2 (3%)
Presence of comorbidities	3 (5%)	0	By calculating proxies for physical susceptibility of being infected Example: weighting for age Z-score [49] or establishing an individual risk model [54]	0
Household income level	5 (8%)	3 (5%)	Results divided by wealth quintile Example: dividing each of the following by Wealth Quintile: Number of deaths averted [56], private expenditure averted [48], or estimated burden due to RV illness [54]	4 (7%)
Relative coverage	18 (30%)	0	Including an adjustment factor for effective coverage Example: Diop et al. [50] divided the coverage in the lowest quintile by the coverage in the entire population	0
Geographic location	16 (27%)	6 (10%)	Results differentiated between rural and urban or divided by state or province Example: deaths averted, OOP expenditure averted and government costs for each rural and urban settings [33]	3 (5%)
Sex at birth	3 (5%)	1 (1.6%)	Input data are differentiated by sex Example: population data by sex, disease incidence and case-fatality rates by sex [53]	0
Loss of productivity	27 (45%)	0	Including costs due to caretakers taking time off from work in the calculation of costs incurred by society	0
Age	26 (43%)	0	DALYs age weighting	0
Financial risk protection (FRP)	3 (5%)	3 (5%)	Through an Extended Cost-Effectiveness analysis Example: calculating a money-metric value of FRP provided by the program [56]	3 (5%)
Reliance on OOP expenditure	35 (58%)	1 (1.6%)	Differentiating the costs incurred by society (societal perspective) Example: calculating the medical and non-medical costs incurred [47]	23 (38%)

^a^n = 60

^b^All the studies having mentioned equity in their study objectives have incorporated at least one indicator in their analysis

## Discussion

An operational set of indicators for formally assessing the types of equity incorporated in cost-effectiveness analysis was developed based on the GPS-Health Equity criteria. This mapping process gathers equity indicators in one checklist. It allows decision-makers to go through functional equity-relevant characteristics and researchers to explore the extent to which equity considerations are captured within the economic evaluation literature. The process of identifying the indicators included a systematic review of the criteria listed in the GPS-Health checklist and review of proposed indicators. The results of this process allows for practical use in assessing published cost-effectiveness analyses. By refining the broad concepts that comprise the criteria, the resulting indicators are foreseen to have two general applications. Firstly, at the decision-making level, the equity indicator mapping provides guidance to policy makers when making resource allocation decisions at the local level. The definition and selection of the appropriate indicators is unlikely to be a one-size-fits-all solution, but the developed indicators provide a start to developing a comprehensive list to select from in light of national health goals. The relevance of each indicator is likely to depend on the context and the specific populations and interventions of interest [58]. For instance, taking into account the presence of comorbidities appeared to be context-specific. Some illnesses might be relevant in one setting but not in another. For example, HIV as comorbidity for childhood rotavirus disease was taken into account in South Africa given the high burden of HIV, while malnutrition was considered as a comorbidity in a Yemen-based analysis. Thus, the list of indicators is a tool that can be used when designing studies that incorporate equity dimensions in economic evaluations. Secondly, for analysts, the indicators can be used to monitor the incorporation of equity in cost-effectiveness analyses, as was done in this case for rotavirus vaccine CEAs. Their application to other diseases and settings is likely to prove useful. It also highlights equity considerations that warrant further evaluation and development, including needed methodological advancement.

The analysis of the set of articles identified through the systematic review distinguished two indicators predominantly included in the existing economic evaluations: severity of illness and age. Severity of illness at the individual level is widely accepted as primary importance to be adopted with the effectiveness of treatment in diverse settings. Use of severity as a priority indicator has been seen in countries such as South Korea [59] and Uganda [60], and has established significance in developed countries through its use in many National Health Services (such as the Norwegian, Finnish, French, Spanish, German and Swedish NHS) [61]. Its major presence (75% of the identified articles) might be due to its relatively easy measurement and link with measurable outcomes with associated costs and health effects. Age was considered through two separate indicators even though its inclusion on equity grounds is still a matter of controversy. Its wide inclusion may reflect the relative ease of incorporating it either via an age distribution function of disease cases and death, or through the age weighting of DALYs. Although age weighting of DALYs, reflecting the higher profile of productivity of young adults, has been criticized and removed from the WHO cost-effectiveness guidelines, its level of use (albeit less frequent) suggests that some researchers might still regard its relevance. Notably, age is also indicative of severity, explaining the high percentage of inclusion of both in CE studies.

Nonetheless, despite the extensive use of these two indicators in the included CEAs, neither indicator was explicitly included in the studies with a specific equity objective tied to these indicators as an equity concern. Few articles formally included equity considerations with distribution purposes. Those that did focused on differences across geographic areas, wealth groups and sex at birth, and considered the impact of the intervention on financial risk protection, and from a societal perspective. They all presented results differentiated according to the indicator, showing the extent to which specific groups would benefit from the vaccine. It is worth mentioning that 30% of those articles having equity as a prime objective followed an extended cost-effectiveness approach, specifically focusing on distributional consequences across distinct strata of populations and medical impoverishment.

When considering health equity indicators, data limitations are relevant, especially when researching low-income countries lacking basic health information systems [13]. Data availability is also likely to affect the choice of indicators.

Several research groups are working on establishing and disseminating new methods, such as ECEA or DCEA, that prescribe combining multiple equity relevant traits into a single social welfare function. However, the literature review findings remain disaggregated per indicator, as no study so far had presented findings in terms of cost-effectiveness of several equity-related indicators together. Results are also contextually bound as the choice of indicators stems from the need of a specific country or country’s wealth level. We noted that few of the included studies simultaneously considered multiple equity criteria. For example, several studies integrating FRP, an important indicator linked to households’ catastrophic health expenditure along with wealth quintiles, omitted the inclusion of severity of illness [15, 48, 56]. A similar matter also applied to geographic location [33, 54] and household income [49] where usually only one dimension was formally incorporated in the analysis. This is likely due to being methodologically demanding by introducing a higher level of complexity to the analysis, which may also be difficult for end users to process. It is also possible that data limitations become more important with the inclusion of multiple indicators, and with the consideration of more indicators, the level of uncertainty in estimates will also increase.

It is important to note that our results are limited by the indicator checklist established and by the case study chosen (CEA of rotavirus vaccines in LMICs). Throughout the research, the inclusion or rejection of some indicators created dilemmas on whether they are, firstly, directly relevant to equity and secondly appropriate to our case study and context. Herd immunity constitutes an example of equity relevance problem. While herd immunity could be considered related to equity if additional protection is conferred upon marginalized groups less likely to be vaccinated, this is not always the case, and following group discussions, this indicator was removed from the list.

As it is unlikely that a single list of indicators would apply uniformly across settings and interventions, these indicators will need to be modified and selected to suit the evaluation of other populations and interventions. Whilst the indicators are likely to have broad relevance for many conditions it is important to note that they were developed specifically for application to this vaccination example.

A further step might be the assessment of other childhood vaccines CEAs in order to monitor and compare the usefulness and applicability of the indicators. Further work can be performed with the list of equity indicators to establish metrics translating the equity dimension as was done with FRP.

## Conclusion

The list of equity indicators developed through this study allows for a systematic assessment of the incorporation of equity dimensions in CEA of childhood vaccines. It also operationalizes Norheim et al.’s GPS-Health checklist. This work highlights the lack of articles which formally include equity considerations with distribution purposes.

Areas warranting consideration on the basis of existing evidence have been highlighted namely: FRP, severity of illness, and reliance on OOP expenditure. Areas necessitating more research have likewise been identified such as presence of comorbidities and additional indirect economic benefits. When many indicators were considered, results were often presented in a disaggregated form. There is a need to develop methodologies reflecting the equity indicator not only in the input but also in the output of an economic evaluation and combining indicators within a single output. A single output might provide more direct comparisons and general conclusions can be drawn taking into account all the subsegments of the population. Nonetheless, this might come at the expense of more nuanced understandings for decision makers.

The identified equity indicators are likely to be useful for the assessment of other childhood vaccines to assess differences in equity consideration. It can also be applied to interventions beyond childhood vaccination, noting that changing the setting might change the focus of the equity dimensions and other dominant indicators might be noted. Our review demonstrates a growing consideration of distributional issues and the expanded use of some indicators in cost-effectiveness analysis.

## Additional files

**Additional file 1: Appendix A.** Norheim Checklist.

**Additional file 2: Appendix B.** Indicators search strategy.

**Additional file 3: Appendix C.** Full data table of results.

**Additional file 4: Appendix D.** **Figure S1.** Trends over time in articles incorporating various equity criteria. **Figure S2.** Average number of indicators included in studies over time.
