# Supplementary material for: Accounting for equity considerations in cost-effectiveness analysis: a systematic review of rotavirus vaccine in low- and middle-income countries
Source: Cost Eff Resour Alloc. 2018 May 18;16:18. doi: 10.1186/s12962-018-0102-2 (PMC5960127; doi:10.1186/s12962-018-0102-2)
Supplement: Supplementary file 1 — Additional file 1: Appendix A. Norheim Checklist. [file 12962_2018_102_MOESM1_ESM.docx]

Appendix A – Norheim Checklist


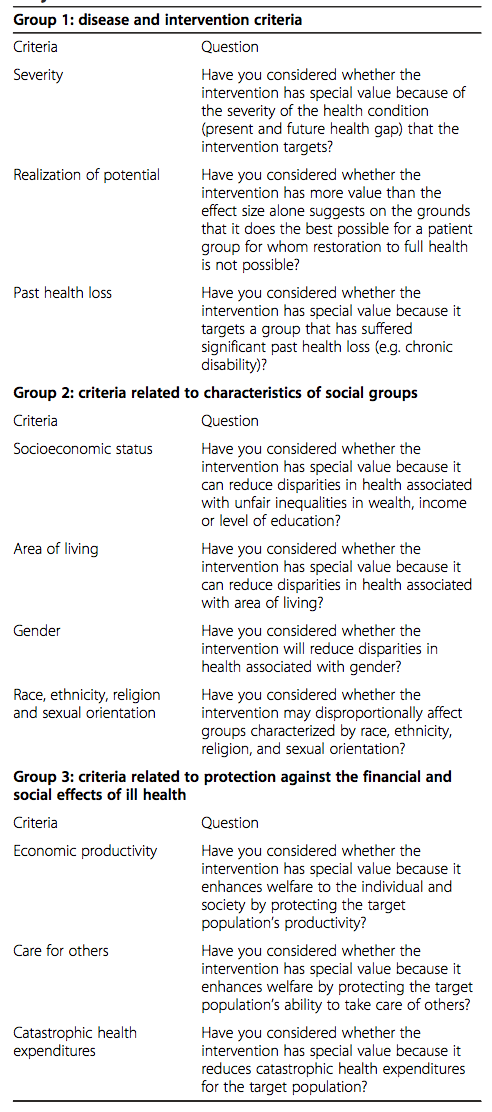


Priority-setting criteria to be considered in conjunction with cost-effectiveness results

From: Norheim OF, Baltussen R, Johri M, Chisholm D, Nord E, Brock D, et al. Guidance on priority setting in health care (GPS-Health): the inclusion of equity criteria not captured by cost-effectiveness analysis. Cost Effectiveness and Resource Allocation. 2014; 12(18).
