# Supplementary material for: Accounting for equity considerations in cost-effectiveness analysis: a systematic review of rotavirus vaccine in low- and middle-income countries
Source: Cost Eff Resour Alloc. 2018 May 18;16:18. doi: 10.1186/s12962-018-0102-2 (PMC5960127; doi:10.1186/s12962-018-0102-2)
Supplement: Supplementary file 2 — Additional file 2: Appendix B. Indicators search strategy. [file 12962_2018_102_MOESM2_ESM.docx]

Appendix B – Indicators search strategy

“severity” AND (“cost-effectiveness” OR “priority setting” OR “decision-making” OR “equity” OR “health care” OR “healthcare”) AND “health”

“realization of potential” AND (“cost-effectiveness” OR “priority setting” OR “decision-making” OR “equity” OR “health care” OR “healthcare”) AND “health”

(“past health loss” OR “past health” OR “health loss”) AND (“cost-effectiveness” OR “priority setting” OR “decision-making” OR “equity” OR “health care” OR “healthcare”) AND “health”

(“socioeconomic” OR “economic status”)AND (“cost-effectiveness” OR “priority setting” OR “decision-making” OR “equity” OR “health care” OR “healthcare”) AND “health”

“area of living” AND (“cost-effectiveness” OR “priority setting” OR “decision-making” OR “equity” OR “health care” OR “healthcare”) AND “health”

“gender” AND (“cost-effectiveness” OR “priority setting” OR “decision-making” OR “equity” OR “health care” OR “healthcare”) AND “health”

“race” AND (“cost-effectiveness” OR “priority setting” OR “decision-making” OR “equity” OR “health care” OR “healthcare”) AND “health”

“ethnicity” AND (“cost-effectiveness” OR “priority setting” OR “decision-making” OR “equity” OR “health care” OR “healthcare”) AND “health”

“religion” AND (“cost-effectiveness” OR “priority setting” OR “decision-making” OR “equity” OR “health care” OR “healthcare”) AND “health”

“sexual orientation” AND (“cost-effectiveness” OR “priority setting” OR “decision-making” OR “equity” OR “health care” OR “healthcare”) AND “health”

(“economic productivity” OR “productivity”) AND (“cost-effectiveness” OR “priority setting” OR “decision-making” OR “equity” OR “health care” OR “healthcare”) AND “health”

“care for others” AND (“cost-effectiveness” OR “priority setting” OR “decision-making” OR “equity” OR “health care” OR “healthcare”) AND “health”

(“catastrophic health expenditure” OR “catastrophic expenditure”) AND (“cost-effectiveness” OR “priority setting” OR “decision-making” OR “equity” OR “health care” OR “healthcare”) AND “health”
