# Supplementary material for: Accounting for equity considerations in cost-effectiveness analysis: a systematic review of rotavirus vaccine in low- and middle-income countries
Source: Cost Eff Resour Alloc. 2018 May 18;16:18. doi: 10.1186/s12962-018-0102-2 (PMC5960127; doi:10.1186/s12962-018-0102-2)
Supplement: Supplementary file 3 — Additional file 3: Appendix C. Full data table of results. [file 12962_2018_102_MOESM3_ESM.docx]

| Appendix C Table | |  | |  | |  | |  | |  | |  | |  | |  | |  | |  | |  | |  | |  | |  | |  | |  | |  | |  | |  | |  | |  | |  | |  | |  | |  | |  | |  | |
| --- | --- | --- | --- | --- | --- | --- | --- | --- | --- | --- | --- | --- | --- | --- | --- | --- | --- | --- | --- | --- | --- | --- | --- | --- | --- | --- | --- | --- | --- | --- | --- | --- | --- | --- | --- | --- | --- | --- | --- | --- | --- | --- | --- | --- | --- | --- | --- | --- | --- | --- | --- | --- | --- | --- | --- |
|  | | | Equity framed in study objective | | Group I | | | | | | | | | | | | Group II | | | | | | | | | | | | | | | | | | | | Group III | | | | | | | | | | | | | | | | Number of countries in the study | |  |
|  | | |  |  | Severity | | | | | | | | Past health loss | | | | Socioeconomic status | | | | | | | | | | | | Area of living | | | | Gender | | | | Economic productivity | | | | | | | | Catastrophic health expenditure | | | | | | | |  |  |  |
|  | | |  |  | Severity of illness at the individual level | | | | Age distribution of the disease | | | | Presence of comorbidities | | | | Household income level | | | | Relative coverage | | | | Education | | | | Geographical access | | | | Sex at birth | | | | Loss of productivity | | | | Age | | | | Financial Risk Protection (FRP) | | | | Reliance on OOP expenditure | | | |  |  |  |
| Author (Year) | | |  |  | M* | | I** | | M | | I | | M | | I | | M | | I | | M | | I | | M | | I | | M | | I | | M | | I | | M | | I | | M | | I | | M | | I | | M | | I | |  |  |  |
| (Abbott et al., 2012) [1] | | | No | | x | | x | |  | |  | |  | |  | |  | |  | | x | |  | |  | |  | |  | |  | |  | |  | | x | |  | |  | |  | |  | |  | | x | |  | | 1 | |  |
| (Ahmeti et al., 2015) [2] | | | No | | x | | x | | x | | x | |  | |  | |  | |  | | x | | x | |  | |  | |  | |  | |  | |  | | x | | x | |  | |  | |  | |  | | x | | x | | 1 | |  |
| (Alkoshi et al., 2014) [3] | | | No | | x | | x | | x | | x | |  | |  | |  | |  | |  | |  | |  | |  | | x | |  | |  | |  | | x | | x | |  | |  | |  | |  | | x | | x | | 1 | |  |
| (Atherly et al., 2012) [4] | | | No | |  | |  | | x | | x | |  | |  | |  | |  | | x | | x | |  | |  | | x | | x | |  | |  | |  | |  | | x | | x | |  | |  | |  | |  | | 74 | |  |
| (Atherly et al., 2009) [5] | | | No | |  | |  | | x | | x | |  | |  | |  | |  | | x | | x | |  | |  | |  | |  | |  | |  | | x | | x | | x | | x | |  | |  | | x | |  | | 72 | |  |
| (Bar-Zeev et al., 2016) [6] | | | No | | x | | x | | x | | x | |  | |  | |  | |  | | x | | x | |  | |  | | x | | x | |  | |  | | x | | x | |  | |  | |  | |  | | x | | x | | 1 | |  |
| (Berry et al., 2010) [7] | | | No | |  | |  | |  | |  | |  | |  | |  | |  | |  | |  | |  | |  | | x | |  | |  | |  | |  | |  | | x | | x | |  | |  | |  | |  | | 1 | |  |
| (Chandrasena et al., 2009) [8] | | | No | | x | |  | | x | | x | |  | |  | |  | |  | |  | |  | |  | |  | | x | | x | |  | |  | | x | | x | |  | |  | | x | |  | | x | | x | | 1 | |  |
| (Chotivitayatarakorn et al., 2010) [9] | | | No | | x | | x | |  | |  | |  | |  | |  | |  | |  | |  | |  | |  | |  | |  | |  | |  | | x | | x | |  | |  | |  | |  | | x | | x | | 1 | |  |
| (Clark et al., 2009) [10] | | | No | | x | | x | | x | | x | |  | |  | |  | |  | | x | | x | |  | |  | | x | | x | |  | |  | | x | |  | | x | | x | |  | |  | | x | | x | | 1 | |  |
| (Constenla et al., 2009) [11] | | | No | | x | | x | | x | | x | | x | |  | |  | |  | | x | |  | |  | |  | | x | | x | |  | |  | | x | |  | | x | | x | |  | |  | | x | |  | | 1 | |  |
| (Constenla et al., 2008) [12] | | | No | | x | | x | | x | | x | |  | |  | |  | |  | | x | |  | |  | |  | |  | |  | |  | |  | | x | | x | | x | | x | |  | |  | | x | | x | | 1 | |  |
| (Constenla et al., 2008) [13] | | | No | | x | | x | |  | |  | |  | |  | |  | |  | |  | |  | |  | |  | |  | |  | |  | |  | | x | | x | |  | |  | |  | |  | | x | | x | | 1 | |  |
| (Constenla et al., 2006) [14] | | | No | | x | | x | |  | |  | |  | |  | |  | |  | |  | |  | |  | |  | |  | |  | |  | |  | | x | |  | |  | |  | |  | |  | | x | |  | | 1 | |  |
| (Constenla et al., 2006) [15] | | | No | | x | | x | | x | | x | |  | |  | | x | |  | |  | |  | |  | |  | |  | |  | |  | |  | | x | | x | |  | |  | |  | |  | | x | | x | | 1 | |  |
| (Cui et al., 2016) [16] | | | No | | x | | x | | x | | x | |  | |  | |  | |  | | x | |  | | x | |  | | x | | x | |  | |  | |  | |  | |  | |  | |  | |  | |  | |  | | 1 | |  |
| (De la Hoz et al., 2010) [17] | | | No | | x | | x | | x | | x | | x | |  | |  | |  | | x | |  | |  | |  | |  | |  | |  | |  | | x | | x | |  | |  | | x | |  | | x | | x | | 1 | |  |
| (De Soàrez et al., 2008) [18] | | | No | |  | |  | |  | |  | |  | |  | |  | |  | |  | |  | |  | |  | | x | |  | |  | |  | | x | | x | |  | |  | |  | |  | | x | | x | | 1 | |  |
| (Diop et al., 2015) [19] | | | No | | x | | x | | x | | x | |  | |  | |  | |  | | x | | x | |  | |  | |  | |  | |  | |  | | x | |  | |  | |  | |  | |  | | x | | x | | 1 | |  |
| (Esposito et al., 2011) [20] | | | No | | x | | x | |  | |  | |  | |  | |  | |  | | x | | x | |  | |  | |  | |  | |  | |  | |  | |  | | x | | x | |  | |  | | x | |  | | 1 | |  |
| (Fischer et al., 2005) [21] | | | No | | x | | x | | x | | x | |  | |  | |  | |  | |  | |  | |  | |  | | x | | x | |  | |  | | x | | x | | x | | x | |  | |  | | x | | x | | 1 | |  |
| (Flem et al., 2009) [22] | | | No | | x | | x | |  | |  | |  | |  | |  | |  | |  | |  | |  | |  | |  | |  | |  | |  | | x | |  | |  | |  | |  | |  | | x | | x | | 1 | |  |
| (Freiesleben de Blasio et al., 2014) [23] | | | No | | x | | x | | x | | x | |  | |  | | x | |  | |  | |  | |  | |  | |  | |  | |  | |  | | x | |  | |  | |  | |  | |  | | x | | x | | 1 | |  |
| (Gargano et al., 2015) [24] | | | No | |  | |  | | x | |  | |  | |  | |  | |  | |  | |  | |  | |  | |  | |  | |  | |  | |  | |  | | x | | x | |  | |  | |  | |  | | 1 | |  |
| (Hacimustafaoglu et al., 2013) [25] | | | No | |  | |  | | x | | x | | x | |  | |  | |  | |  | |  | |  | |  | |  | |  | |  | |  | | x | |  | |  | |  | |  | |  | | x | |  | | 1 | |  |
|  |  | | |  | |  | |  | |  | |  | |  | |  | |  | |  | |  | |  | |  | |  | |  | |  | |  | |  | |  | |  | |  | |  | |  | |  | |  | |  | |  | |
| Table 2 |  | | |  | |  | |  | |  | |  | |  | |  | |  | |  | |  | |  | |  | |  | |  | |  | |  | |  | |  | |  | |  | |  | |  | |  | |  | |  | |  | |
|  | | | Equity framed in study objective | | Group I | | | | | | | | | | | | Group II | | | | | | | | | | | | | | | | | | | | Group III | | | | | | | | | | | | | | | | Number of countries in the study | |  |
|  | | |  |  | Severity | | | | | | | | Past health loss | | | | Socioeconomic status | | | | | | | | | | | | Area of living | | | | Gender | | | | Economic productivity | | | | | | | | Catastrophic health expenditure | | | | | | | |  |  |  |
|  | | |  |  | Severity of the disease at the individual level | | | | Age distribution of the disease | | | | Presence of comorbidities | | | | Household income level | | | | Relative coverage | | | | Education | | | | Geographical access | | | | Sex at birth | | | | Loss of Productivity | | | | Age | | | | Financial Risk Protection (FRP) | | | | Reliance on OOP expenditure | | | |  |  |  |
| Author (Year) | | |  |  | M* | | I** | | M | | I | | M | | I | | M | | I | | M | | I | | M | | I | | M | | I | | M | | I | | M | | I | | M | | I | | M | | I | | M | | I | |  |  |  |
| (Isakbaeva et al., 2006) [26] | | | No | | x | | x | |  | |  | |  | |  | |  | |  | |  | |  | |  | |  | |  | |  | |  | |  | | x | | x | | x | | x | | x | |  | | x | | x | | 1 | |  |
| (Javanbakht et al., 2015) [27] | | | No | | x | | x | | x | | x | |  | |  | |  | |  | | x | | x | |  | |  | | x | | x | |  | |  | |  | |  | |  | |  | |  | |  | | x | | x | | 1 | |  |
| (John et al., 2014) [28] | | | No | | x | | x | |  | |  | |  | |  | |  | |  | |  | |  | |  | |  | | x | |  | |  | |  | |  | |  | |  | |  | |  | |  | | x | | x | | 1 | |  |
| (Kim et al., 2011) [29] | | | Yes | | x | |  | | x | | x | |  | |  | |  | |  | |  | |  | |  | |  | |  | |  | |  | |  | |  | |  | | x | | x | |  | |  | | x | |  | | 72 | |  |
| (Kim et al., 2009) [30] | | | No | | x | | x | | x | | x | |  | |  | |  | |  | |  | |  | |  | |  | |  | |  | |  | |  | | x | | x | | x | |  | |  | |  | | x | | x | | 1 | |  |
| (Koksal et al., 2016) [31] | | | No | | x | | x | |  | |  | |  | |  | |  | |  | |  | |  | |  | |  | |  | |  | |  | |  | | x | |  | |  | |  | |  | |  | | x | |  | | 1 | |  |
| (Liu et al., 2012) [32] | | | No | | x | | x | | x | | x | |  | |  | |  | |  | |  | |  | |  | |  | |  | |  | |  | |  | | x | | x | | x | | x | | x | |  | | x | | x | | 1 | |  |
| (Martí et al., 2015) [33] | | | No | | x | | x | | x | | x | |  | |  | |  | |  | | x | | x | |  | |  | |  | |  | |  | |  | | x | | x | |  | |  | |  | |  | | x | | x | | 1 | |  |
| (Megiddo et al., 2014) [34] | | | Yes | |  | |  | | x | | x | |  | |  | | x | | x | | x | | x | | x | |  | | x | | x | | x | | x | |  | |  | | x | | x | |  | |  | |  | |  | | 1 | |  |
| (Mousavi Jarravi et al., 2016) [35] | | | No | | x | | x | | x | | x | |  | |  | |  | |  | |  | |  | |  | |  | |  | |  | |  | |  | | x | | x | | x | | x | |  | |  | | x | | x | | 1 | |  |
| (Ortega et al., 2009) [36] | | | No | | x | | x | |  | |  | | x | |  | |  | |  | |  | |  | |  | |  | | x | | x | |  | |  | | x | |  | | x | | x | |  | |  | | x | |  | | 1 | |  |
| (Patel et al., 2013) [37] | | | No | | x | | x | | x | | x | | x | |  | |  | |  | | x | |  | |  | |  | | x | |  | |  | |  | |  | |  | | x | | x | |  | |  | | x | |  | | 1 | |  |
| (Paternina-Caicedo et al., 2015) [38] | | | No | | x | | x | | x | | x | |  | |  | |  | |  | |  | |  | |  | |  | |  | |  | |  | |  | | x | |  | | x | |  | |  | |  | | x | |  | | 116 | |  |
| (Pecenka et al., 2015) [39] | | | Yes | |  | |  | | x | | x | | x | | x | | x | | x | | x | | x | |  | |  | | x | |  | |  | |  | |  | |  | |  | |  | | x | | x | | x | | x | | 1 | |  |
| (Podewils et al., 2005) [40] | | | Yes | | x | | x | | x | | x | |  | |  | |  | |  | |  | |  | |  | |  | | x | |  | |  | |  | | x | |  | | x | | x | |  | |  | | x | |  | | Asian countries | |  |
| (Rheigans et al., 2007) [41] | | | No | | x | | x | | x | | x | |  | |  | |  | |  | |  | |  | |  | |  | |  | |  | |  | |  | | x | | x | | x | | x | |  | |  | | x | |  | | 8 | |  |
| (Rheingans et al., 2014) [42] | | | Yes | | x | | x | | x | | x | | x | | x | | x | | x | | x | | x | |  | |  | | x | | x | | x | | x | |  | |  | | x | | x | |  | |  | |  | |  | | 1 | |  |
| (Rheingans et al., 2012) [43] | | | Yes | |  | |  | | x | | x | | x | | x | | x | | x | | x | | x | | x | |  | | x | | x | | x | |  | |  | |  | |  | |  | |  | |  | |  | |  | | 25 | |  |
| (Rheingans et al., 2009) [44] | | | No | |  | |  | | x | | x | |  | |  | |  | |  | | x | | x | | x | |  | |  | |  | |  | |  | | x | | x | | x | | x | |  | |  | | x | | x | | Developing countries | |  |
| (Rose et al., 2009) [45] | | | No | | x | | x | |  | |  | |  | |  | |  | |  | |  | |  | |  | |  | |  | |  | |  | |  | | x | | x | | x | | x | |  | |  | | x | | x | | 1 | |  |
| (Ruhago et al., 2015) [46] | | | Yes | | x | | x | | x | | x | |  | |  | | x | |  | |  | |  | |  | |  | | x | | x | |  | |  | | x | |  | | x | |  | |  | |  | | x | |  | | 1 | |  |
| (Shakerian et al., 2015) [47] | | | No | | x | | x | | x | | x | |  | |  | |  | |  | |  | |  | |  | |  | |  | |  | |  | |  | | x | | x | |  | |  | |  | |  | | x | | x | | 1 | |  |
| (Sigei et al., 2015) [48] | | | No | | x | | x | | x | | x | |  | |  | |  | |  | | x | | x | |  | |  | |  | |  | |  | |  | |  | |  | |  | |  | |  | |  | | x | | x | | 2 | |  |
| (Smith et al., 2011) [49] | | | No | | x | | x | |  | |  | | x | |  | | x | |  | | x | | x | |  | |  | | x | | x | |  | |  | |  | |  | |  | |  | |  | |  | | x | |  | | 1 | |  |
|  |  | | |  | |  | |  | |  | |  | |  | |  | |  | |  | |  | |  | |  | |  | |  | |  | |  | |  | |  | |  | |  | |  | |  | |  | |  | |  | |  | |
|  |  | | |  | |  | |  | |  | |  | |  | |  | |  | |  | |  | |  | |  | |  | |  | |  | |  | |  | |  | |  | |  | |  | |  | |  | |  | |  | |  | |
| Table 2 |  | | |  | |  | |  | |  | |  | |  | |  | |  | |  | |  | |  | |  | |  | |  | |  | |  | |  | |  | |  | |  | |  | |  | |  | |  | |  | |  | |
|  | | | Equity framed in study objective | | Group I | | | | | | | | | | | | Group II | | | | | | | | | | | | | | | | | | | | Group III | | | | | | | | | | | | | | | | Number of countries in the study | |  |
|  | | |  |  | Severity | | | | | | | | Past health loss | | | | Socioeconomic status | | | | | | | | | | | | Area of living | | | | Gender | | | | Economic productivity | | | | | | | | Catastrophic health expenditure | | | | | | | |  |  |  |
|  | | |  |  | Severity of the disease at the individual level | | | | Age distribution of the disease | | | | Presence of comorbidities | | | | Household income level | | | | Relative coverage | | | | Education | | | | Geographical access | | | | Sex at birth | | | | Loss of Productivity | | | | Age | | | | Financial Risk Protection (FRP) | | | | Reliance on OOP expenditure | | | |  |  |  |
| Author (Year) | | |  |  | M* | | I** | | M | | I | | M | | I | | M | | I | | M | | I | | M | | I | | M | | I | | M | | I | | M | | I | | M | | I | | M | | I | | M | | I | |  |  |  |
| (Sun et al., 2016) [50] | | | No | | x | | x | | x | | x | |  | |  | |  | |  | |  | |  | |  | |  | |  | |  | |  | |  | | x | | x | | x | | x | |  | |  | | x | | x | | 1 | |  |
| (Tate et al., 2011) [51] | | | No | | x | | x | | x | | x | |  | |  | |  | |  | | x | | x | |  | |  | |  | |  | |  | |  | | x | |  | | x | | x | |  | |  | | x | |  | | 1 | |  |
| (Tate et al., 2009) [52] | | | No | | x | | x | |  | |  | |  | |  | |  | |  | |  | |  | |  | |  | | x | | x | |  | |  | | x | | x | | x | | x | |  | |  | | x | | x | | 1 | |  |
| (Tu et al., 2012) [53] | | | No | | x | | x | | x | | x | |  | |  | |  | |  | |  | |  | |  | |  | |  | |  | |  | |  | | x | | x | |  | |  | |  | |  | | x | | x | | 1 | |  |
| (Uruena et al., 2015) [54] | | | Yes | | x | | x | | x | | x | |  | |  | | x | |  | | x | | x | | x | |  | | x | | x | |  | |  | | x | | x | | x | |  | |  | |  | | x | | x | | 1 | |  |
| (Valencia-Mendoza et al., 2008) [55] | | | No | | x | | x | | x | | x | | x | |  | | x | |  | |  | |  | |  | |  | |  | |  | |  | |  | | x | |  | |  | |  | |  | |  | | x | |  | | 1 | |  |
| (Van Hoek et al., 2012) [56] | | | No | | x | | x | | x | | x | | x | |  | |  | |  | |  | |  | |  | |  | |  | |  | |  | |  | |  | |  | | x | | x | |  | |  | |  | |  | | 1 | |  |
| (Verguet et al., 2015) [57] | | | Yes | | x | |  | | x | |  | |  | |  | | x | |  | |  | |  | | x | |  | | x | |  | |  | |  | | x | |  | |  | |  | | x | | x | | x | | x | | 1 | |  |
| (Verguet et al., 2013) [58] | | | Yes | |  | |  | | x | |  | |  | |  | | x | | x | |  | |  | |  | |  | | x | |  | |  | |  | | x | |  | |  | |  | | x | | x | | x | | x | | 2 | |  |
| (Wang et al., 2009) [59] | | | No | |  | |  | | x | | x | |  | |  | |  | |  | |  | |  | |  | |  | | x | |  | |  | |  | | x | | x | |  | |  | |  | |  | | x | | x | | 1 | |  |
| (Wilopo et al., 2009) [60] | | | No | | x | | x | | x | | x | |  | |  | |  | |  | |  | |  | |  | |  | |  | |  | | x | | x | | x | | x | | x | | x | |  | |  | | x | | x | | 1 | |  |

*M: Mentioned: an “x” under the column labeled “M” would indicate that the indicator was mentioned in the study (whether in the introduction, methods, results, discussion or conclusion)

**I: Included: an “x” under the column labeled “I” would indicate that the indicator was taken into account in the study

**References**

1. Abbott C, Tiede B, Armah G, Mahmoud A. Evaluation of cost-effectiveness of live oral pentavalent reassortant rotavirus vaccine introduction in Ghana. Vaccine. 2012;30(15):2582-2587.

2. Ahmeti A, Preza I, Simaku A, Nelaj E, Clark AD, Felix Garcia AG, et al. Cost-effectiveness of rotavirus vaccination in Albania. Vaccine. 2015;33(Suppl 1)A201-208.

3. Alkoshi S, Maimaiti N, Dahlui M. Cost-effectiveness analysis of rotavirus vaccination among Libyan children using a simple economic model. Libyan Journal of Medicine. 2014;9(1):26236.

4. Atherly DE, Lewis KD, Tate J, Parashar UD, Rheingans RD. Projected health and economic impact of rotavirus vaccination in GAVI-eligible countries: 2011-2030. Vaccine. 2012;30(Suppl 1):A7-14.

5. Atherly D, Dreibelbis R, Parashar UD, Levin C, Wecker J, Rheingans RD. Rotavirus vaccination: cost-effectiveness and impact on child mortality in developing countries. J Infect Dis. 2009;200(Suppl 1):S28-38.

6. Bar-Zeev N, Tate JE, Pecenka C, Chikafa J, Mvula H, Wachepa R, et al. Cost-effectiveness of monovalent rotavirus vaccination of infants in Malawi: a postintroduction analysis using individual patient-level costing data. Clin Infect Dis. 2016;62(Suppl 2):S220-228.

7. Berry SA, Johns B, Shih C, Berry AA, Walker DG. The cost-effectiveness of rotavirus vaccination in Malawi. J Infect Dis. 2010;202(Suppl):S108-115.

8. Chandrasena N, Rajindrajith S, Ahmed K, Pathmeswaran A, Nakagomi O. Hospital-based study of the severity and economic burden associated with rotavirus diarrhea in Sri Lanka. Journal of Pediatric Infectious Diseases. 2015;4(4):379-386.

9. Chotivitayatarakorn P, Chotivitayatarakorn P, Poovorawan Y. Cost-effectiveness of rotavirus vaccination as part of the national immunization program for Thai children. Southeast Asian Journal of Tropical Medicine and Public Health. 2010;41(1).

10. Clark AD, Walker DG, Mosqueira NR, Penny ME, Lanata CF, Fox-Rushby J, et al. Cost-effectiveness of rotavirus vaccination in peru. J Infect Dis. 2009;200(Suppl1):S114-124.

11. Constenla D, Velásquez FR, Rheigans RD, Antil L, Cervantes Y. Economic impact of a rotavirus vaccination program in Mexico. Pan Am J Public Health. 2009;25(6).

12. Constenla DO, Linhares AC, Rheingans RD, Antil LR, Waldaman EA, da Silva LJ. Economic impact of rotavirus vaccination in Brazil. Health Popul Nutr. 2008;26(4):388-396.

13. Constenla D, Ortega-Barría E, Rheigans RD, Antil L, Sáez-Lorens X. Economic impact of rotavirus vaccination in Panamá. An Pediatr (Barc). 2008;68(2):128-135.

14. Constenla D, O'Ryan M, Navarrete MS, Antil L, Rheingans RD. Potential cost effectiveness of a rotavirus vaccine in Chile. Revista médica de Chile. 2006;134:679-688.

15. Constenla D, Pérez-Schael I, Rheigans RD, Antil L, Salas H, Yarzábal JP. Assessment of the economic impact of the antiretroviral vaccine in Venezuela. Pan Am J Public Health. 2006;20(4).

16. Cui S, Tobe RG, Mo X, Liu X, Xu L, Li S. Cost-effectiveness analysis of rotavirus vaccination in China. Projected possibility of scale-up from the current domestic option. BMC Infect Dis. 2016;16(1):677.

17. De la Hoz F, Alvis N, Narváez J, Cediel N, Gamboa O, Velandia M. Potential epidemiological and economical impact of two rotavirus vaccines in Colombia. Vaccine. 2010;28(22):3856-3864.

18. de Soárez PC, Valentim J, Sartori AMC, Novaes HMD. Cost-effectiveness analysis of routine rotavirus vaccination in Brazil. Pan Am J Public Health. 2009;23(4).

19. Diop A, Atherly D, Faye A, Lamine Sall F, Clark AD, Nadiel L, et al. Estimated impact and cost-effectiveness of rotavirus vaccination in Senegal: A country-led analysis. Vaccine. 2015;33(Suppl 1):A119-125.

20. Esposito DH, Tate JE, Kang G, Parashar UD. Projected impact and cost-effectiveness of a rotavirus vaccination program in India, 2008. Clin Infect Dis. 2011;52(2):171-177.

21. Fischer TK, Anh DD, Antil L, Cat NDL, Kilgore PE, Thiem VD, et al. Health care costs of diarrheal disease and estimates of the cost-effectiveness of rotavirus vaccination in Vietnam. Journal of Infectious Diseases. 2005;192:1720-1726.

22. Flem ET, Latipov R, Nurmatov ZS, Xue Y, Kasymbekova KT, Rheingans RD. Costs of diarrheal disease and the cost-effectiveness of a rotavirus vaccination program in kyrgyzstan. J Infect Dis. 2009;200(Suppl 1):S195-202.

23. Freiesleben de Blasio B, Flem E, Latipov R, Kuatbaeva A, Kristiansen IS. Dynamic modeling of cost-effectiveness of rotavirus vaccination, Kazakhstan. Emerg Infect Dis. 2014;20(1):29-37.

24. Gargano LM, Tate JE, Parashar UD, Omer SB, Cookson ST. Comparison of impact and cost-effectiveness of rotavirus supplementary and routine immunization in a complex humanitarian emergency, Somali case study. Confl Health. 2015;9:5.

25. Hacimustafaoglu M, Celebi S, Akin L, Agin M, Sevencan F. Cost Effectiveness of both (Monovalent and Pentavalent) rotavirus vaccines. Çocuk Enfeksiyon Dergisi/Journal of Pediatric Infection. 2013;13(1):13-20.

26. Isakbaeva ET, Musabaev E, Antil L, Rheingans R, Juraev R, Glass RI, et al. Rotavirus disease in Uzbekistan: cost-effectiveness of a new vaccine. Vaccine. 2007;25(2):373-380.

27. Javanbakht M, Moradi-Lakeh M, Yaghoubi M, Esteghamati A, Mansour Ghanaie R, Mahmoudi S, et al. Cost-effectiveness analysis of the introduction of rotavirus vaccine in Iran. Vaccine. 2015;33(Suppl 1):A192-200.

28. John J, Sarkar R, Muliyil J, Bhandari N, Bhan MK, Kang G. Rotavirus gastroenteritis in India, 2011-2013: revised estimates of disease burden and potential impact of vaccines. Vaccine. 2014;32(Suppl 1):A5-9.

29. Kim SY, Sweet S, Chang J, Goldie SJ. Comparative evaluation of the potential impact of rotavirus versus HPV vaccination in GAVI-eligible countries: a preliminary analysis focused on the relative disease burden. BMC Infect Dis. 2011;11:174.

30. Kim SY, Goldie SJ, Salomon JA. Cost-effectiveness of Rotavirus vaccination in Vietnam. BMC Public Health. 2009;9:29.

31. Koksal T, Akelma AZ, Koksal AO, Kutukoglu I, Ozdemir O, Yuksel CN, et al. Cost-effectiveness of rotavirus vaccination in Turkey. J Microbiol Immunol Infect. 2017;50(5):693-699.

32. Liu N, Yen C, Fang ZY, Tate JE, Jiang B, Parashar UD, et al. Projected health impact and cost-effectiveness of rotavirus vaccination among children <5 years of age in China. Vaccine. 2012;30(48):6940-6945.

33. Marti SG, Alcaraz A, Valanzasca P, McMullen M, Standaert B, Garay U, et al. Cost effectiveness evaluation of a rotavirus vaccination program in Argentina. Vaccine. 2015;33(42):5684-5690.

34. Megiddo I, Colson AR, Nandi A, Chatterjee S, Prinja S, Khera A, et al. Analysis of the Universal Immunization Programme and introduction of a rotavirus vaccine in India with IndiaSim. Vaccine. 2014;32(Suppl 1):A151-161.

35. Mousavi Jarrahi Y, Zahraei SM, Sadigh N, Esmaeelpoor Langeroudy K, Khodadost M, Ranjbaran M, et al. The cost effectiveness of rotavirus vaccination in Iran. Hum Vaccin Immunother. 2016;12(3):794-800.

36. Ortega O, El-Sayed N, Sanders JW, Abd-Rabou Z, Antil L, Bresee J, et al. Cost-benefit analysis of a rotavirus immunization program in the Arab Republic of Egypt. J Infect Dis. 2009;200(Suppl 1):S92-98.

37. Patel HD, Roberts ET, Constenla DO. Cost-effectiveness of a new rotavirus vaccination program in Pakistan: a decision tree model. Vaccine. 2013;31(51):6072-6078.

38. Paternina-Caicedo A, De la Hoz-Restrepo F, Alvis-Guzman N. Epidemiological and economic impact of monovalent and pentavalent rotavirus vaccines in low and middle income countries: a cost-effectiveness modeling analysis. Pediatr Infect Dis J. 2015;34(7):e176-184.

39. Pecenka CJ, Johansson KA, Memirie ST, Jamison DT, Verguet S. Health gains and financial risk protection: an extended cost-effectiveness analysis of treatment and prevention of diarrhoea in Ethiopia. BMJ Open. 2015;5(4):e006402.

40. Podewils LJ, Antil L, Hummelman E, Bresee J, Parashar UD, Rheignas R. Projected cost-effectiveness of rotavirus vaccination for children in Asia. Journal of Infectious Diseases. 2005;192(Suppl 1):S133.

41. Rheingans R, Constenla D, Antil L, Innis BL, Breuer T. Potential cost-effectiveness of vaccination for rotavirus gastroenteritis in eight Latin American and Caribbean countries. Pan Am J Public Health. 2007;21(4).

42. Rheingans R, Anderson JD, Anderson B, Chakraborty P, Atherly D, Pindolia D. Estimated impact and cost-effectiveness of rotavirus vaccination in India: effects of geographic and economic disparities. Vaccine. 2014;32(Suppl 1):A140-150.

43. Rheingans R, Atherly D, Anderson J. Distributional impact of rotavirus vaccination in 25 GAVI countries: estimating disparities in benefits and cost-effectiveness. Vaccine. 2012;30(Suppl 1):A15-23.

44. Rheingans RD, Antil L, Dreibelbis R, Podewils LJ, Bresee JS, Parashar UD. Economic costs of rotavirus gastroenteritis and cost-effectiveness of vaccination in developing countries. J Infect Dis. 2009;200(Suppl 1):S16-27.

45. Rose J, Hawthorn RL, Watts B, Singer ME. Public health impact and cost effectiveness of mass vaccination with live attenuated human rotavirus vaccine (RIX4414) in India: model based analysis. BMJ. 2009;339:b3653.

46. Ruhago GM, Ngalesoni FN, Robberstad B, Norheim OF. Cost-effectiveness of live oral attenuated human rotavirus vaccine in Tanzania. Cost Eff Resour Alloc. 2015;13:7.

47. Shakerian S, Moradi Lakeh M, Esteghamati A, Zahraei M, Yaghoubi M. Cost-effectiveness of rotavirus vaccination for under-five children in Iran. Iran J Pediatr. 2015;25(4):e2766.

48. Sigei C, Odaga J, Mvundura M, Madrid Y, Clark AD, Kenya ProVac Technical Working Group, et al. Cost-effectiveness of rotavirus vaccination in Kenya and Uganda. Vaccine. 2015;33(S):A109-A118.

49. Smith ER, Rowlinson EE, Iniguez V, Etienne KA, Rivera R, Mamani N, et al. Cost-effectiveness of rotavirus vaccination in Bolivia from the state perspective. Vaccine. 2011;29(38):6704-6711.

50. Sun SL, Gao YQ, Yin J, Zhuang GH. A cost-effectiveness analysis on universal infant rotavirus vaccination strategy in China. Chinese Journal of Epidemiology. 2016;37(2).

51. Tate JE, Kisakye A, Mugyenyi P, Kizza D, Odiit A, Braka F. Projected health benefits and costs of pneumococcal and rotavirus vaccination in Uganda. Vaccine. 2011;29(17):3329-3334.

52. Tate JE, Rheingans RD, O'Reilly CE, Obonyo B, Burton DC, Tornheim JA, et al. Rotavirus disease burden and impact and cost-effectiveness of a rotavirus vaccination program in Kenya. J Infect Dis. 2009;200(Suppl 1):S76-84.

53. Tu HA, Rozenbaum MH, Coyte PC, Li SC, Woerdenbag HJ, Postma MJ. Health economics of rotavirus immunization in Vietnam: potentials for favorable cost-effectiveness in developing countries. Vaccine. 2012;30(8):1521-1528.

54. Uruena A, Pippo T, Betelu MS, Virgilio F, Hernandez L, Giglio N, et al. Cost-effectiveness analysis of rotavirus vaccination in Argentina. Vaccine. 2015;33(Suppl 1):A126-134.

55. Valencia-Mendoza A, Bertozzi SM, Gutierrez JP, Itzler R. Cost-effectiveness of introducing a rotavirus vaccine in developing countries: the case of Mexico. BMC Infect Dis. 2008;8:103.

56. van Hoek AJ, Ngama M, Ismail A, Chuma J, Cheburet S, Mutonga D, et al. A cost effectiveness and capacity analysis for the introduction of universal rotavirus vaccination in Kenya: comparison between Rotarix and RotaTeq vaccines. PLoS One. 2012;7(10):e47511.

57. Verguet S, Olson ZD, Babigumira JB, Desalegn D, Johansson KA, Kruk ME, et al. Health gains and financial risk protection afforded by public financing of selected interventions in Ethiopia: an extended cost-effectiveness analysis. The Lancet Global Health. 2015;3(5):e288-e296.

58. Verguet S, Murphy S, Anderson B, Johansson KA, Glass R, Rheingans R. Public finance of rotavirus vaccination in India and Ethiopia: an extended cost-effectiveness analysis. Vaccine. 2013;31(42):4902-4910.

59. Wang XY, Riewpaiboon A, von Seidlein L, Chen XB, Kilgore PE, Ma JC, et al. Potential cost-effectiveness of a rotavirus immunization program in rural China. Clin Infect Dis. 2009;49(8):1202-1210.

60. Wilopo SA, Kilgore P, Kosen S, Soenarto Y, Aminah S, Cahyono A, et al. Economic evaluation of a routine rotavirus vaccination programme in Indonesia. Vaccine. 2009;27(Suppl 5):F67-74.
