# Supplementary material for: Accounting for equity considerations in cost-effectiveness analysis: a systematic review of rotavirus vaccine in low- and middle-income countries
Source: Cost Eff Resour Alloc. 2018 May 18;16:18. doi: 10.1186/s12962-018-0102-2 (PMC5960127; doi:10.1186/s12962-018-0102-2)
Supplement: Supplementary file 4 — Additional file 4: Appendix D. Figure S1. Trends over time in articles incorporating various equity criteria. Figure S2. Average number of indicators included in studies over time. [file 12962_2018_102_MOESM4_ESM.docx]

Appendix D -

Figure S1: Trends over time in articles incorporating various equity criteria

Proportion* of articles incorporating A. Severity of the disease, B. Age distribution, C. Presence of comorbidities, D. Household income level, E. Sex at birth, F. Loss of productivity, G. Financial Risk Protection, H. Reliance on OOP expenditure

* Percentage of articles out of total number of articles published during the specified period of time

Figure S2: Average number of indicators included in studies over time
